# DIGITAL MONITORING OF SLEEP, MEALS, AND EXERCISE AS A PREVENTIVE INTERVENTION FOR DEPRESSION IN BEREAVED SPOUSES

**DOI:** 10.1093/geroni/igz038.2249

**Published:** 2019-11-08

**Authors:** Sarah Stahl

**Affiliations:** University of Pittsburgh, Pittsburgh, Pennsylvania, United States

## Abstract

The death of a spouse brings profound change to bereaved survivors’ lifestyle and daily routine. These changes disrupt circadian rhythms which, in turn, places individuals at high risk for depression. The purpose of this study is to examine the feasibility and acceptability of a 12-week behavioral intervention that targets the timing and regularity of sleep, meals, and physical activity via digital monitoring and motivational health coaching. Participants were 60+ years of age and assessed on intervention acceptability and adherence, depression symptoms (Hamilton Rating Scale for Depression) and the rest-activity rhythm, (a downstream indicator of the body’s circadian rhythm (via actigraphic technology). The intervention was rated highly by participants (n=55); 88% were compliant in digital monitoring and 95% were retained. Depression symptoms declined from pre-to post-intervention; and the regularity of circadian rhythms increased. An intervention that targets the regularity of day- and nighttime activities may reduce depression in older spousally-bereaved adults.

